# Antioxidant, LC-MS Analysis, and Cholinesterase Inhibitory Potentials of *Phoenix dactylifera* Cultivar Khudari: An In Vitro Enzyme Kinetics and In Silico Study

**DOI:** 10.3390/biom13101474

**Published:** 2023-09-30

**Authors:** Sami G. Almalki, Yaser E. Alqurashi, Wael Alturaiki, Saud Almawash, Amir Khan, Parvej Ahmad, Danish Iqbal

**Affiliations:** 1 Department of Medical Laboratory Sciences, College of Applied Medical Sciences, Majmaah University, Majmaah 11952, Saudi Arabia; w.alturaiki@mu.edu.sa; 2 Department of Biology, College of Science Al-Zulfi, Majmaah University, Al-Majmaah 11952, Saudi Arabia; y.alqurashi@mu.edu.sa; 3 Department of Pharmaceutical Sciences, College of Pharmacy, Shaqra University, Shaqra 11961, Saudi Arabia; salmawash@su.edu.sa; 4 Oral Medicine and Allied Dental Sciences Department, Faculty of Dentistry, Taif University, Taif 11099, Saudi Arabia; amirkhan@tudent.org; 5 IIRC-5 Clinical Biochemistry and Natural Product Research Laboratory, Integral University, Lucknow 226026, India; contactpahmad@gmail.com; 6 Department of Health Information Management, College of Applied Medical Sciences, Buraydah Private Colleges, Buraydah 51418, Saudi Arabia

**Keywords:** antioxidant, acetylcholinesterase, enzyme inhibition kinetics, LC/MS analysis, molecular dynamics simulation, *Phoenix dactylifera*

## Abstract

We evaluated the therapeutic potentials of Khudari fruit pulp, a functional food and cultivar of *Phoenix dactylifera*, against neurological disorders. Our results demonstrate a good amount of phytochemicals (total phenolic content: 17.77 ± 8.21 µg GA/mg extract) with a high antioxidant potential of aqueous extract (DPPH assay IC_50_ = 235.84 ± 11.65 µg/mL) and FRAP value: 331.81 ± 4.56 µmol. Furthermore, the aqueous extract showed the marked inhibition of cell-free acetylcholinesterase (*electric eel*) with an IC_50_ value of 48.25 ± 2.04 µg/mL, and an enzyme inhibition kinetics study revealed that it exhibits mixed inhibition. Thereafter, we listed the 18 best-matched phytochemical compounds present in aqueous extract through LC/MS analysis. The computational study revealed that five out of eighteen predicted compounds can cross the BBB and exert considerable aqueous solubility. where 2-{5-[(1E)-3-methylbuta-1,3-dien-1-yl]-1H-indol-3-yl}ethanol (MDIE) indicates an acceptable LD_50_. value. A molecular docking study exhibited that the compounds occupied the key residues of acetylcholinesterase with ΔG range between −6.91 and −9.49 kcal/mol, where MDIE has ∆G: −8.67 kcal/mol, which was better than that of tacrine, ∆G: −8.25 kcal/mol. Molecular dynamics analyses of 100 ns supported the stability of the protein–ligand complexes analyzed through RMSD, RMSF, Rg, and SASA parameters. TRP_84 and GLY_442 are the most critical hydrophobic contacts for the complex, although GLU_199 is important for H-bonds. Prime/MM-GBSA showed that the protein–ligand complex formed a stable confirmation. These findings suggest that the aqueous extract of Khudari fruit pulp has significant antioxidant and acetylcholinesterase inhibition potentials, and its compound, MDIE, forms stably with confirmation with the target protein, though this fruit of Khudari dates can be a better functional food for the treatment of Alzheimer’s disease. Further investigations are needed to fully understand the therapeutic role of this plant-based compound via in vivo study.

## 1. Introduction

Alzheimer’s disease (AD) stands as one of the top three contributors of neurological disorders mainly associated with old age, with around 130,000 cases reported only in Saudi Arabia [1]. The symptoms of AD patients generally include memory loss, agitation, apathy, cognitive impairments, and aberrant motor behavior [2]. The pathophysiology of AD is quite complex, and studies have reported that amyloid-beta (AB) deposition forms plaques, and the hyperphosphorylation of tau proteins forms neurofibrillary tangles during AD, which further disturbs redox balance and the cholinergic activity of neurons [3,4,5]. Normally, cholinergic activity greatly depends on the release of acetylcholine (ACh), a neurotransmitter that further binds to ACh receptors and transmits the signals, or it can be hydrolyzed via acetylcholinesterase (AChE) [6]. It was reported that, during AD, due to neuronal damage or the degradation of cholinergic neuron-rich region, there is a sudden decline in ACh levels, and cholinesterase enzymes further decrease its levels, which hinders the neuronal signal transmission [7]. The elevated level of acetylcholine in synapses stimulates nicotinic and muscarinic receptors, which give beneficial relief for cognitive problems in AD [8,9]. Therefore, cholinesterase inhibition can manage AD by reducing the catalysis of Ach, which ultimately supports the signal’s transmission and improves the life quality of AD patients.

However, the most common FDA-approved medication for AD, i.e., cholinesterase inhibitors (tacrine, donepezil), exhibits several side effects, such as nausea, headache, decrease appetite, and vomiting [10,11]. As a result, there is a great deal of interest in developing more effective treatments to alleviate illness symptoms and stop the loss of neurons, which causes the disease. Natural ingredients and their byproducts are known to have higher therapeutic potentials for a variety of metabolic and infectious problems, including diabetes, oxidative damage, high cholesterol levels, ulcer, neurological disorders, tumors, and infections caused by microbes [12,13,14,15,16,17,18,19,20,21,22]. Because nature provides a plentiful supply of bioactive metabolites, it may be safe and cost-effective to develop new inhibitors of important regulatory enzymes for the treatment of metabolic diseases, including neurological illnesses [15,23].

The date palm (*Phoenix dactylifera* L.) represents the most grown fruit-bearing cultivated crop in the world’s arid and semiarid areas. Throughout the millennia, Islamic physicians have carried out plenty of research related to *Phoenix dactylifera* in the treatment and management of various disease, as they believed that the Prophet Mohammed (Peace Be Upon Him) said: “*There is no disease that Allah has created, except that Allah also has created its cure”* [24]. *Phoenix dactylifera* leaves, fruit, pits, and pollen have been utilized in the management of various health problems and disorders because they are a significant source of bioactive chemicals that are primarily responsible for their therapeutic activity [25]. Fruits of *Phoenix dactylifera* are high in carbs, amino acids, vitamins, and minerals. Alkaloids, tannins, glycosides, flavonoids, steroids, terpenoids, and carotenoids are active phytoconstituents in fruits. The fruit’s principal active ingredients, including flavonoids, exhibit various medicinal properties. The plant extract also contains antioxidants and has neuroprotective properties, and the fruits have the potential to help control Alzheimer’s disease [26]. Khudari, a cultivar of *Phoenix dactylifera* that is mostly cultivated in the Al-Qassim region, possesses a higher protein content (3.42 gm/100 gm dry weight), higher energy values (287.5 kcal/100 gm), and a lower amount of lipids (0.18 gm/100 gm DW) than most *Phoenix dactylifera* varieties [27,28]. The total phenol content (TPC) and radical scavenging (DPPH) potentials of extracts of Khudari fruits were found to be nearly identical to Awja date varieties (the most researched and therapeutic variety of dates). This date (Khudari) was also shown to have a significantly reduced level of sugar (74.5 grammes/100 gm DW) [29,30].

Various natural bioactive compounds have been identified in fruit extract of *Phoenix dactylifera*, i.e., luteolin, protocatechuic acid, quercetin, gallic acid, resorcinol, chelidonic acid, and chlorogenic acid [31]. The antioxidant potential of *Phoenix dactylifera* is possible due to the occurrence of phenolics, melatonin, carotenoids, and vitamins contents, e.g., p-coumaric acid [32]. Hepatoprotective activity and protection against elevated alkaline phosphatase was also observed, and this is due to the presence of bioactive compounds i.e., ferulic acid, caffeic acid, quercetin, anthocyanins, and proanthocyanidins [33,34]. There have been plenty of in silico, in vitro, and in vivo reports published, which exhibit the potential effect of fruit extract and their bioactive compounds in the treatment and management of AD via modulating various risk factors, i.e., antioxidant and hepatoprotective activity, memory and behavior improvement, and cerebral anti-ischemic, neuroprotective, anxiolytic, nootropic, and antipsychotic activities [35,36,37]. There is no study available that demonstrates the inhibitory activity of AChE from *Phoenix dactylifera* fruits-derived bioactive compounds. Therefore, we selected *Phoenix dactylifera* fruit pulp extract and its bioactive compounds as a potential AChE inhibitor for investigation. 

It is generally known that computational biology and in vitro inhibition of enzymes are the primary techniques for elucidating binding patterns and optimizing bioactive chemicals for target-based drug development [38,39,40,41,42]. However, despite having beneficial phytoconstituents and antioxidant activity, this Khudari variety of dates is still the least explored for its therapeutic potentials against several pathologies. Considering these facts, we planned to explore the therapeutic potential of the fruit pulp of Khudari cultivars of *Phoenix dactylifera* and their secondary metabolites for the treatment of neurological disorders through cholinesterase inhibition via a variety of methods, such as cell-free enzyme inhibition kinetics, molecular docking, and MDS (molecular dynamics simulation) studies.

## 2. Materials and Methods

Chemicals: The solvent methanol (MeOH) was procured from Thermo Fisher Scientific Pvt. Ltd. Waltham, MA, USA, while 1,1-diphenyl-2-picrylhydrazyl (DPPH), ascorbic acid, 2,4,6-tripyridyl-s-triazine (TPTZ), ferric chloride (FeCl_3_), Folin–Ciocalteu, and ferrous sulphate (FeSo_4_) were purchased from Scientific Laboratory Supplies, Nottingham, UK. In addition to the above-mentioned chemicals, tacrine hydrochloride and acetylcholinesterase as analytical-grade chemicals were procured from Sigma Aldrich, Burlington, MA, USA.

### 2.1. Collection and Preparation of Dates Extract

Khudari cultivars of *Phoenix dactylifera* dates were procured from a local market of the Qassim region in Saudi Arabia. The fruit pulp of dates (500 g) was separated, dried in a shed, and pulverized into powder form. The powdered form of the date weighed around 25 gm and was extracted using the optimum amount (1:10 ratio) of methanol and water solvent in the Soxhlet apparatus (Borosil), respectively, until the solvent became colorless. Once the solvent attained room temperature, it was filtered and air-dried, and the residue was scraped out and subsequently stored at −20 °C for further experimental use.

The method for calculating percent yield of different fractions is as follows:

$$\%yield=\frac{Dry\;weight\;of\;plant\;extract}{Dry\;weight\;of\;plant\;material}\times 100$$

### 2.2. Total Phenolic Content (TPC) and Phytochemical Analysis

Khudari date extracts were tested qualitatively for phytochemical composition using a standard approach [43]. To measure the total phenolic content of the extracts, the Folin–Ciocalteu method was used [44].

### 2.3. Antioxidant Property Assay

#### 2.3.1. Scavenging Activity of DPPH Radical

The methanolic and aqueous fraction of Khudari extracts were scrutinized for their potential to scavenge the DPPH radical using Brand-William’s method. Briefly, 3 mL of DPPH solution was added to 100 µL of plant extract and the reference drug (ascorbic acid) and incubated at 37 °C for 30 min; thereafter, absorbance was measured at 517 nm with a Biospectrum kinetics spectrophotometer (Eppendorf) [45]. To measure the percentage (%) of DPPH free radicals that were scavenged, the following equation was applied:

$$\%DPPH=\frac{\Delta Absorbance\;of\;control-\Delta absorbance\;of\;test\;sample}{\Delta Absorbance\;of\;control}\times 100$$

#### 2.3.2. Analysis of Ferric-Reducing Antioxidant Power

An adaptation of Benzie and Strain’s method was used for assessment of the ferric-reducing antioxidant potential (FRAP) of Khudari extracts [46]. Concisely, A freshly prepared FRAP reagent can be attained by combining sodium acetate buffer (300 mM, pH 3.6), 10 mM TPTZ solution, and 20 mM FeCl_3_ solution in a volume ratio of 10:1:1. To 3 mL of FRAP reagent, 100 microliters of sample were added at varying concentrations. The absorbance was determined at 593 nm after incubating it for 30 min at room temperature. Results were expressed as µmol Fe (II)/g dry weight of date material, based on plots of the standard curve using the FeSO_4_ solution.

### 2.4. Cell-Free Enzyme Inhibition Assay

#### 2.4.1. Acetylcholinesterase Inhibition Assay

We performed the acetylcholinesterase test according to Ellman’s protocol with slight modifications [47]. In a 2 mL cuvette, 33 μL of 10 mM-DTNB, 100 μL of 1 mM-AChI, and 767 μL of 50 mM-Tris HCl (calibrated at pH = 8.0), along with 100 μL of extracts at different concentrations, were added. The above-mentioned cuvette was blank, whereas in another cuvette, 300 μL of buffer was substituted with an equal volume of 0.28 U ml^−1^ of acetylcholinesterase enzyme. To carry out the competitive analysis, standard drug tacrine was used. The following reaction was monitored continuously in a Biospectrum kinetics spectrophotometer (Eppendorf) for 20 min at 405 nm wavelength. The experiment was performed in triplicate. The following equation was used to estimate the percentage (%) of acetylcholinesterase enzyme activity:

$$\%inhibition=\frac{\Delta Absorbance\;of\;control-\Delta Absorbance\;of\;sample}{\Delta Absorbance\;of\;control}\times 100$$

#### 2.4.2. Spectrometric Study of the Enzyme Inhibition Kinetics

Kinetic analysis was carried out at 30 °C using four distinct concentrations (0.5, 1.0, 1.5, and 2.0 mM) of substrate (AChI) to determine the AChE inhibition mechanism via absence/presence of various inhibitory concentrations of standard (tacrine) and aqueous fractions of Khudari extract. “AChE catalyzed-hydrolysis” of AChI was analyzed kinetically using spectrophotometry (Eppendorf) at a wavelength of 405 nm for a total of 20 min, and the OD values were measured at 60 s intervals. Additionally, a Lineweaver-Burk, Dixon, and Km and V_max_ plot was devised to define the inhibitory mechanism [48].

#### 2.4.3. LC-MS (Liquid Chromatography–Mass Spectrometry) Analysis

The fraction that exhibited the greatest inhibition of AChE activity was further explored for bioactive compounds through LC-MS analysis, performed at the Sophisticated Analytical Instrument Facility, CSIR-Central Drug Research Institute, Lucknow. The LC-MS and MS/MS simulations were carried out using a Waters Alliance E2695/HPLC-TQD mass spectrometer with a mass range of 100–2000 Da. Separation was achieved on an Agilent Poroshell 120 EC C-18 column (150 mm × 4.6 mm, 2.7 μm). Two mobile phases were used: A-0.1% formic acid in water and B-90% of acetonitrile in water, at a flow rate of 1.500 mL/min. The gradient started with A-5% and B-95%, a linear increase in A% and decrease in B% after 6 min at A-30% and B-70%; during 12–16 min, A-60% B-40%; 20 min, A-80% and B-20%; and 26–30, min A-5% and B-95%. The chromatograms were compared to the available literature to identify the peaks, and then, the output file was subjected to analyze the peaks via Mnova software version 14.2.1 offered by Mestrelab Research, chemistry software solutions.

### 2.5. Computational Study

#### 2.5.1. Platform of In Silico Computational Study

The computer-added drug design studied was performed on a Lenovo Notebook equipped with Intel(R) Core (TM) i5-8265U @1.80 GHz powered machine with 2 GB graphics card (GeForce MX250 NVIDIA) and 8 GB of RAM. The software for molecular modelling studies, such as AutoDock 4.2 32-bit (http://autodock.scripps.edu/ (accessed on 16 April 2023)), Cygwin, SPDBV, Discovery studio (DS) visualizer, ChemDraw, Notepad++ version 7.9.2., and Open Babel GUI, was installed. The Desmond (Schrodinger-2020, LLC, New York, NY, USA) tool was used for the molecular dynamics simulation study, carried out on workstation equipped with NVIDIA Quadro P5000 graphic card, 28 GB RAM, and 3.50 GHz processor.

#### 2.5.2. Retrieval and Preparation of 3D Structure of Ligands

The LC-MS-predicted compound’s 3D structures were retrieved through the *PubChem* (http://pubchem.ncbi.nlm.nih.gov (accessed on 16 May 2023)) database as an SDF file, which was later converted to a PDB file format using Discovery studio visualizer, and the energy was minimized by applying CHARMM force field using the steepest descent method for 500 steps and an RMS gradient of 0.01. Thereafter, the ligands were converted to a “.pdbqt” file format [42,49]. 

#### 2.5.3. Retrieval and Preparation of Target Protein

The target protein 3D structure of AChE (ID: 1ACJ) was downloaded from the protein data bank (PDB) (https://www.rcsb.org/search (accessed on 16 May 2023)) and visualized by Discovery Studio Visualizer 2020 (https://discover.3ds.com/discovery-studio-visualizer-download (accessed on 3 October 2020)) [50]. The resolution of the selected AChE crystal structure was 2.80 Å. The active site of the protein was obtained through an online tool, the Play-Molecule (https://www.playmolecule.com/ (accessed on 16 May 2023)) DEEPSITE [51]. The structure of the target protein was cleaned by removal of heteroatoms and polar hydrogen and addition of Kollmann charges using AutoDock tools [52]. After that, the final structure for docking was prepared by converting it to a PDBQT file format through AutoDock 4.2 tool [42,52,53,54].

#### 2.5.4. Molecular Docking Studies of Selected Ligands against AChE

Molecular docking was performed by using the AutoDock 4.2 version as described earlier [55,56]. Briefly, Hetatms were removed, and Kollman charges were assigned to protein (1ACJ), and the processed protein was saved in a “.pdbqt” file for docking study. The grid box size was maintained at 60 × 60 × 60 points (x, y, and z, respectively) with 0.608 Å grid spacing, and the grid center was selected at dimensions x, y, and z: −7.36, 64.04, and 134.87, respectively. A total of forty independent docking runs were executed for each ligand. The conformational clustering of the poses of the target–ligand complexes was determined by considering a root mean square deviation tolerance <2.0 Å. The complexes (target–ligand) with the least free energy of binding (ΔG) were considered the most stable and indicated the inhibitory effects of ligands [53,57]. Further, the protein–ligand complex interaction was studied using Discovery studio visualizer version 2020.

#### 2.5.5. Pharmacokinetics and Drug-Likeness Profiling of Ligands

All the predicted compounds (ligands) were evaluated for their pharmacokinetics and physicochemical parameters via SWISS ADME tool [58]. They were also evaluated for their drug-like characteristics based on their agreement with Lipinski’s rule of five (http://www.swissadme.ch (accessed on 18 May 2023)) [59].

#### 2.5.6. Predicted Toxicity Assessment of the Selected Ligands

The ProTox-II web-server (https://tox-new.charite.de/protox_II/index.php?site=compound_input (accessed on 18 May 2023)), an online tool that predicts the toxicity of small organic molecules, was used for the toxicity assessment. It provides extensive facts concerning the toxicity of the chemicals, such as LD_50_, carcinogenicity, immunotoxicity, mutagenicity, cytotoxicity, and hepatotoxicity [60].

#### 2.5.7. Molecular Dynamics Simulation Analysis

The complex of the best-scoring ligand (best hit) and AChE protein (PDB id: 1ACJ) was selected for the molecular dynamics simulation study using Schrodinger-2020, LLC, New York, NY, USA tools [42]. The complex was imported to Maestro interface for pre-processing (optimization and minimization).

The protein–ligand complex developed with AutoDock Vina was imported into the Maestro interface in Schrodinger’s software 2020. The optimization and minimization of the protein–ligand complex was performed using Maestro’s Protein Processing Wizard. All the systems were built using the System Builder tool. As a solvent model with an orthorhombic box (10 Å), TIP3P was selected. The simulation used the OPLS_2005 force field [61]. The models were neutralized by using counterions. The addition of sodium chloride (NaCl) 0.15 M simulated physiological conditions. The normal pressure test (NPT) ensemble with a pressure of one atmosphere and 300 K temperature was employed for the whole simulation. The trajectories were saved for analysis every 100 ps. Measures including the root mean square deviation (RMSD), the percentage of secondary structural components, root mean square fluctuation (RMSF), and protein–ligand interactions were analyzed to assess the stability of the protein–ligand complexes. The findings of the three independent research studies are shown as mean ± standard deviation.

#### 2.5.8. Calculations of Free Energy (Prime-MM/GBSA)

Using the Prime module (Schrodinger, LLC, New York, NY, USA) and the MM-GBSA approach, the interaction’s free energy value for the protein–ligand complex was calculated as previously described [42]. Once equilibration was achieved, free energy was estimated on every 10 ns MD simulation trajectory up to 100 ns. In conclusion, the docked complexes were first optimized locally in Prime using molecular mechanics (MM), and then, their energies were reduced using the OPLS-AA (2005) force field and the generalized Born surface area (GBSA) continuum solvent model.

## 3. Results and Discussion

### 3.1. Phytochemical Estimation of Khudari Fruit Pulp

Our results showed the qualitative presence/absence of different phytochemicals, such as alkaloids, carbohydrate, flavonoids, glycosides, tannins, terpenoids, phenols, cardiac glycosides, and steroids, in both the MeOH and aqueous fraction of Khudari extract (Table 1). The percentage yield was found to be MeOH 48%, and aqueous 36%. Phytochemicals are easily accessible, bioavailable, and far less toxic than synthetic medications and show significant promise for preventing and treating AD. They also have several synergistic benefits, such as improving cognitive and cholinergic functioning. A great amount of scientific research has demonstrated the anti-Alzheimer’s disease benefits of polyphenols, including cell damage protection, neuroprotective effects, AChE inhibitory action, and amyloid aggregation [62,63].

### 3.2. Scavenging Property of DPPH Radical

**Figure 1:**
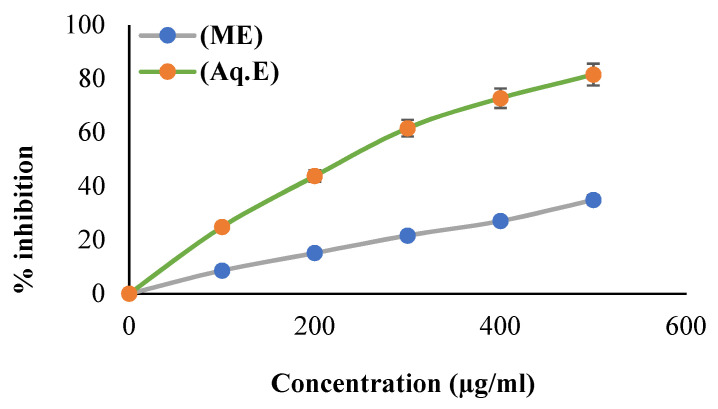
Radical (DPPH) scavenging potentials of aqueous and methanolic extract of Khudari fruit pulp. The data are represented as mean ± S.D. in triplicate value.

The comparatively stable DPPH radical is commonly employed to assess the free radical scavenging ability of natural antioxidants such as fruit and plant extracts. The percent scavenging capacity of DPPH radical by the MeOH and aqueous fraction of Khudari extract is shown in Figure 1. The strongest antioxidant activity was found in the aqueous fraction (IC_50_ value: 235.84 ± 11.24 µg/mL) in a dose-dependent style, where the ascorbic acid used as reference standard showed an IC_50_ value of 15.58 ± 0.76 µg/mL. Our results are in agreement with previous reports of free radical scavenging potentials of Khudari fruit extract [28,29].

### 3.3. Ferric-Ions-Reducing Potential and Total Phenolic Content

**Figure 2:**
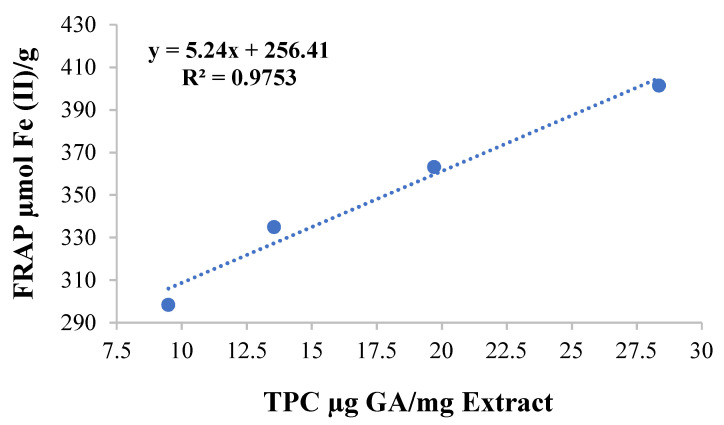
Correlation graph of TPC and FRAP values of aqueous extract of Khudari fruit pulp.

The FRAP (ferric-reducing antioxidant property) assay was performed to evaluate the total antioxidant capacity of the Khudari extract fractions. The assay is based on the extract’s ability to reduce ferric ions to ferrous ones. Our findings elucidated that the aqueous fraction has significantly higher values of FRAP than the MeOH, which are 331.81 ± 4.56 µmol and 51.57 ± 1.183 µmol Fe (II)/g, respectively. The total content of phenols of both extracts was measured by the standard-procedure Folin–Ciocalteu method. The result shows that the highest content of phenols was found in the aqueous (17.77 ± 8.21 µg GA/mg extract) rather than the MeOH (7.70 ± 0.45 µg GA/mg extract) fraction. A linear relationship test was also used to examine the relationship between TPC and FRAP. Our findings indicated that as the phenolic content rose, the ferric-reducing potential also increased. Based on our data, we can conclude that TPC and FRAP have a positive association (Figure 2).

### 3.4. AChE Inhibition Property and Mode of Inhibition

**Figure 3:**
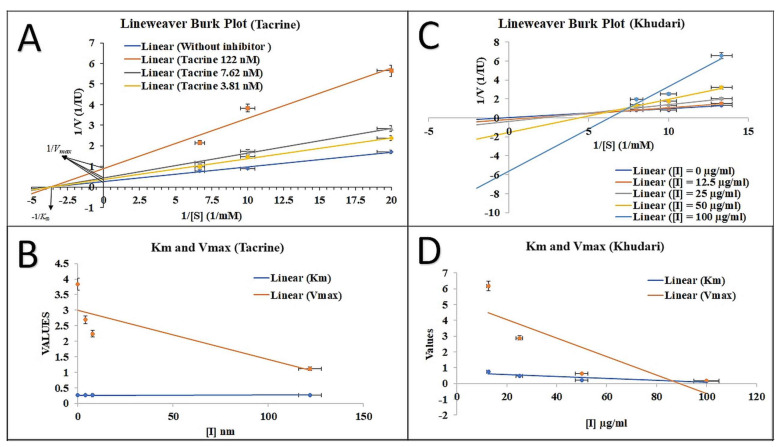
Lineweaver–Burk double reciprocal and K*_m_* and V_max_ plot of 1/v versus 1/[S] of (**A**,**B**) tacrine and (**C**,**D**) Khudari aqueous extract against AChE.

Based on the antioxidant property of the aqueous fraction, we further examined its AChE inhibitory activity. The aqueous fraction of Khudari extract showed the marked inhibition of AChE in a dose-dependent manner, with an IC_50_ value of 48.25 ± 2.04 µg/mL. We observed 3.59% inhibition at 12.5 µg/mL, and the percentage inhibition increased to 34.72% at 25 µg/mL and 51.28% at 50 µg/mL, whereas percentage inhibition for the highest concentration used, which was 100 µg/mL, was almost 66.26% inhibition of AChE activity. The reference standard showed an IC_50_ value of 17.31 nM. We further determined the mode of inhibition (enzyme inhibition kinetic) of the aqueous fraction via the AChI-hydrolysis inhibition at different concentrations (Figure 3A–D). The standard drug tacrine binds to the allosteric site, a site that differs from the active site of the enzyme, resulting in decreased enzyme affinity without competing with its substrate binding site. The fundamental method for distinguishing non-competitive inhibition from competitive inhibition is by the decrease in Vmax and the unchanged Km (Figure 3A, B). On the other hand, enzyme kinetic studies revealed that the aqueous fraction of Khudari extracts at different concentrations do not intersect on any axis, nor is their slope same; thus, they exhibit a mixed type of inhibition that might be attributed to the presence of several secondary metabolites in the plant extract (Figure 3C). Considering the decline in both the values of K*_m_* and V_max_ by increasing the dose concentration, it is evident that the plant extract exhibits non-competitive inhibition. The plant extract is composed of several secondary metabolites, and therefore, some of them are involved in a non-competitive mode of inhibition and some in a mixed type of inhibition (competitive and non-competitive) (Figure 3D). Therefore, from the data, we can clearly state that there was a mixed type of AChE inhibition demonstrated by the aqueous fraction. Our findings are consistent with earlier reports in which tacrine was described as a reversible non-competitive inhibitor towards the AChE enzyme [64,65] as well as a mixed-type inhibitor with a high non-competitive component [66].

### 3.5. LC/MS Analysis for Identification of Phytochemical Compounds in Aqueous Fraction of Phoenix dactylifera (Khudari Cultivar)

In the present work, bioactive composition from the aqueous fraction of fruit pulp of *Phoenix dactylifera* (Khudari cultivar) was carried out on a Waters Alliance E2695/HPLC-TQD mass spectrometer in positive- and negative-ionization mode. The output file was analyzed in the Mnova software offered by Mestrelab Research, chemistry software solutions, and the peaks were matched by using the inbuilt molecular match plugin tool. The reference compounds for the molecular match analysis were downloaded from the *PubChem* classification browser> spectral information> Mass Spectrometry> LC-MS, sub-category, in which 18,804 compounds were enlisted. According to the Mnova analysis, a total of the 18 best-matched phytochemical compounds are listed in Table 2 and Table 3.

The compounds identified in this study were tentatively characterized using MS data, and the understanding of the observed MS/MS spectra as compared with reference compounds was obtained from *PubChem* using Mnova software.

**Figure 4:**
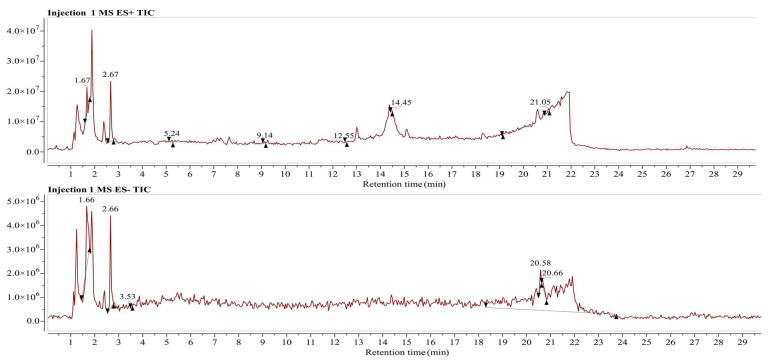
Chromatograms of LC-MS data.

The positive- and negative-ionization modes were used to identify the compounds in the aqueous fraction, with five parameters: molecular weight, retention duration, *m/z* [M − H]^−^, *m/z* [M − H]^+^, molecular formula, and fragmentation pattern [67]. The representations of phytochemicals in the form of chromatograms from LC-MS data are shown in Figure 4.

**Figure 5:**
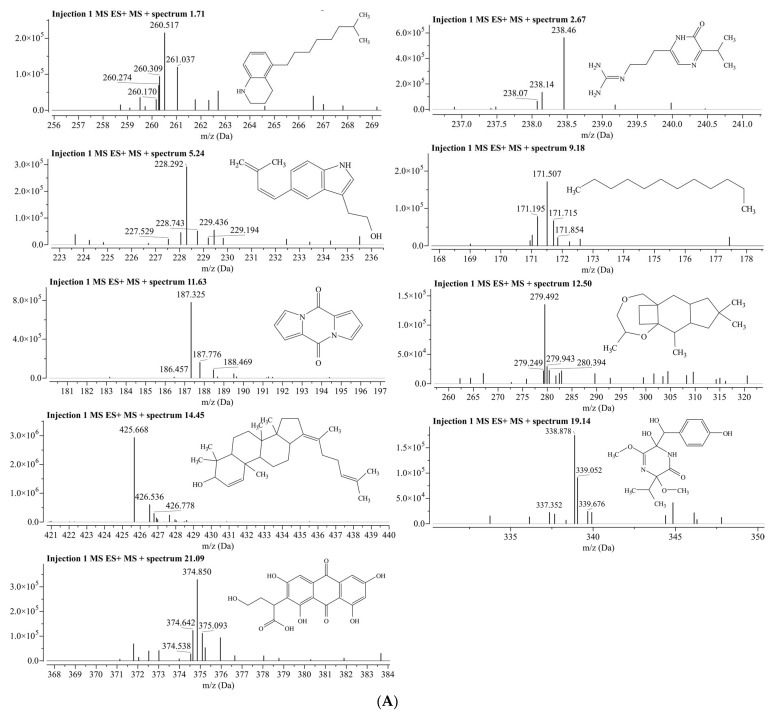

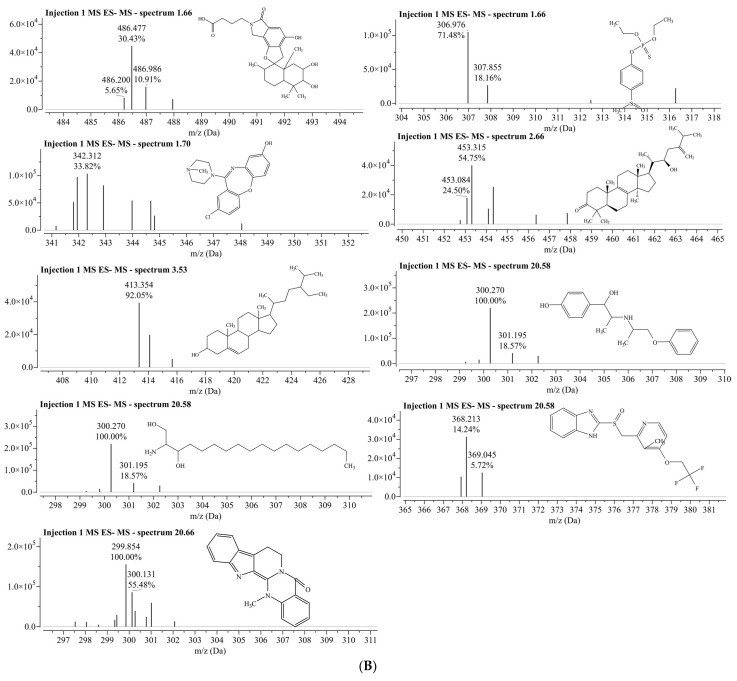
(**A**) Positive ionization. (**B**) Negative ionization.

The positive-ion [M − H]+ chromatograms peaks showed the presence of nine compounds, i.e., 5-(7-Methyloctyl)-1,2,3,4-tetrahydroquinoline (MS peak at *m/z* 260.5 and retention time Rt at 1.71 min); Argvalin (*m/z* 238.4, and Rt 2.67 min); 2-{5-[(1E)-3-methylbuta-1,3-dien-1-yl]-1H-indol-3-yl}ethanol (*m/z* 228.2, and Rt 5.24); Dodecane (*m/z* 171.5, Rt 9.18); Pyrocoll (*m/z* 187.3 and Rt 11.63); Methyl hydroxysterpurate ethylidene acetal (*m/z* 279, and Rt 12.50); 3β-hydroxy-4β-methylfusida-17(20)(16,21-cis),24-diene (*m/z* 425.6 and Rt 14.45); Terezine E (*m/z* 338.8 and Rt 19.14); and Paeciloquinone D (*m/z* 374.8 and Rt 21.09) (Figure 5A). Meanwhile, in the negative-ion mode [M − H]^−^ chromatograms, nine compounds were detected, i.e., 4-[(2R,3S,7R,8R,8aS) -2,3,4′-trihydroxy-4,4,7, 8a-tetramethyl-6′-oxospiro [2,3,4a,5,6,7, hexahydro-1H-naphthalene-8,2′-3,8-dihydrofuro [2,3-e]isoindole]-7′-yl]butanoic acid (*m/z* 486.4, and Rt 1.66); Fensulfothion (*m/z* 306.9, and Rt 1.66); 8-Hydroxyloxapine (*m/z* 343.3 Rt 1.70); Gilvsin A (*m/z* 453.3 and Rt 2.66); Sitosterol (*m/z* 413.3 and Rt 3.53); Lansoprazole (*m/z* 368.2 and Rt 20.58); Isoxsuprine (*m/z* 300.2 and Rt 20.58); 2-Aminooctadecane (*m/z* 300.2 and Rt 20.58); and Dehydroevodiamine (*m/z* 299.8 and Rt 20.66) (Figure 5B).

### 3.6. ADME Pharmacokinetic Profiling of the Detected Compounds

**Figure 6:**
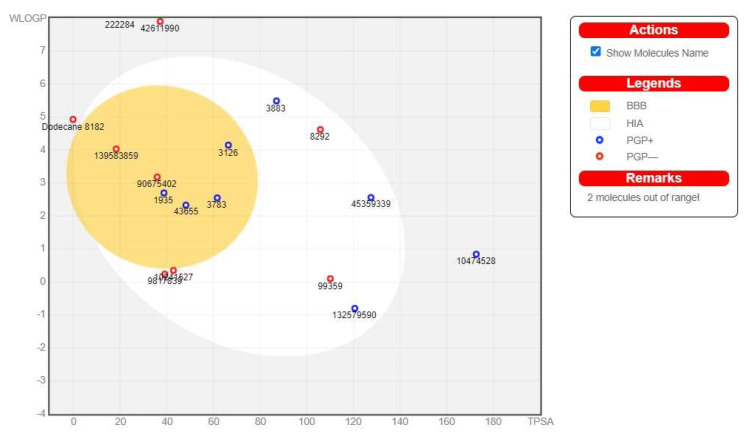
The boiled-egg representation of the selected compounds. Five compounds were in the yellow region, i.e., *PubChem* IDs 3126, 3783, 43655, 90675402, and 139583859, indicating that they can cross the BBB.

Our target is AChE, which primarily maintains the neurotransmitter homeostasis via hydrolyzing the acetylcholine (Ach) into acetic acid and choline. Neuronal activity is directly connected with the brain, so the oral bioactive compounds must penetrate the blood–brain barrier (BBB) to attain their activity. The pharmacological measurements, as well as the BBB-penetration power of chemicals via using computational methods, are the most effective and plausible steps in the field of drug discovery [68]. The BBB-penetration power was predicted by the ADME tool in the form of a boiled egg, where white region denoted the probability of gastrointestinal absorption, and yellow (yolk) showed the BBB-passing capability [69]. According to the SWISS ADME analysis, there were five predicted compounds (2-Aminooctadecane, Isoxsuprine, 8-Hydroxyloxapine, 2-{5-[(1E)-3-methylbuta-1,3-dien-1-yl]-1H-indol-3-yl} ethanol (MDIE), and Methyl hydroxysterpurate ethylidene acetal (MHEA)) within the yolk region, which means they can cross the BBB. The absorption capacity across the BBB was high in 2-Aminooctadecane, Isoxsuprine, and MDIE but moderate in 8-Hydroxyloxapine and MHEA [70,71] (Figure 6).

All five compounds exert considerable aqueous solubility, ranging from 2.32 to 629.45 mg/L. The permeability of Caco-2 and MDCK cells has been identified as a critical parameter in the drug development process [71,72,73]. The pre-ADME analysis evaluated the permeability score of Caco-2 and MDCK cells, ere ranging from 15.32–57.68 nm/s and 0.23 to 231.82 nm/s, respectively. The appropriate range for the permeability score of Caco-2 and MDCK cells is classified as low (<4 nm/s), moderate (>4–70 nm/s), and high (>70 nm/s). [74]. Additionally, the skin permeability (Log Kp) of these compounds was also assessed and found appropriate for the drug candidate, ranging from −0.77 to −3.75 cm/h [75]. Plasma protein binding (PPB) was found to be the most important parameter for distribution of the drug from the plasma to the target tissue, which ultimately effects the drug efficacy [76,77]. Our compounds showed different efficiencies of PPB, in which 2-Aminooctadecane and Methyl hydroxysterpurate ethylidene acetal (MHEA) were found to have the highest PPB efficiency, that is, 100% (Table 4). It is commonly known that drug diffusion is inversely proportional to the efficiency of PPB. We can clearly see that the higher the PPB efficiency, the lower the drug diffusion, and the lower the PPB efficiency, the higher the drug diffusion throughout the body.

Similarly, human intestinal absorption (HIA) and cytochrome P450-2D6 (CYP 2D6) are responsible for the drug absorption and excretion in the body [78,79,80]. Our results demonstrated that two compounds, namely 2-Aminooctadecane and Isoxsuprine, showed inhibitory activity against CYT P450 2D6, while the rest were non-inhibitors. The inhibition of the CYTP450 2D6 via these compounds results in increased bioavailability, and clearance from circulation was also delayed. This may eventually lead to increased therapeutic efficacy of these drugs. Contrarily, CYT P450 2D6 was shown as a substrate by three compounds: 2-Aminooctadecane, Isoxsuprine, and 8-Hydroxyloxapine. This reflects their rapid metabolism, immediate therapeutic activities, and clearance from the body.

### 3.7. Drug-Likeness Properties of Predicted Compounds

A set of distinct factors is involved in the assessment of the drug-likeness property of a chemical entity. To date, there are several in silico approaches used to access the drug-likeness property to prevent wasting time, money, and labor; hence, we used a computational tool to analyze the ADMET parameters [68,81]. Our selected compounds were tested for the same to assess Lipinski’s rule of five (RO5) and the physiochemical and drug-like properties. The RO5 is a guideline for determining whether a chemical molecule with a specific pharmacological or biological activity possesses qualities that would make it a likely orally active medication in humans [82]. The chemical entity to be utilized as a medicine for oral delivery, if a biologically active molecule, must meet five requirements. Poor absorption or permeation are most likely if the molecular weight >500 Da, hydrogen bond donors >5, hydrogen bond acceptor > 10, and logP (octanol water partition coefficient) >5 [82]. An orally active drug-like compound should have no more than one violation of the following criteria as stated by RO5. Based on the drug-likeness analysis, our selected compounds obeyed all the parameters of Lipinski’s rule of five except one compound, i.e., 2-Aminooctadecane, which violated one Lipinski’s rule (LogP > 5). However, this violation is not enough to exclude this compound from the study (Table 5). These characteristics indicate that the chosen compounds passed all the drug-likeness tests and could therefore be evaluated for additional pharmacological actions.

### 3.8. Assessment of Toxicity of Selected Compounds

Toxicology evaluation using AI-based methods is a vital step in the drug discovery process that helps to cut down on time, resources, labor, and animal experiments. The toxicity of the selected ligands was assessed by an online web server, i.e., ProToxII. The server predicted the hepatotoxicity, carcinogenicity, immunogenicity, mutagenicity, as well as cytotoxicity along with LD_50_. The output results are tabulated in Table 6.

Our results indicated that 2-Aminooctadecane and MHEA successfully qualified all the parameters of toxicity, while MDIE indicated suitable LD_50_ but is active against immunotoxicity. On the other hand, Isoxsuprine and 8-Hydroxyloxapine (toxic against immune system) showed low LD_50_, falling under class III and class II categories, respectively.

### 3.9. The Selected Five Compounds Strongly Bind to the Active Pocket of AChE

**Figure 7:**
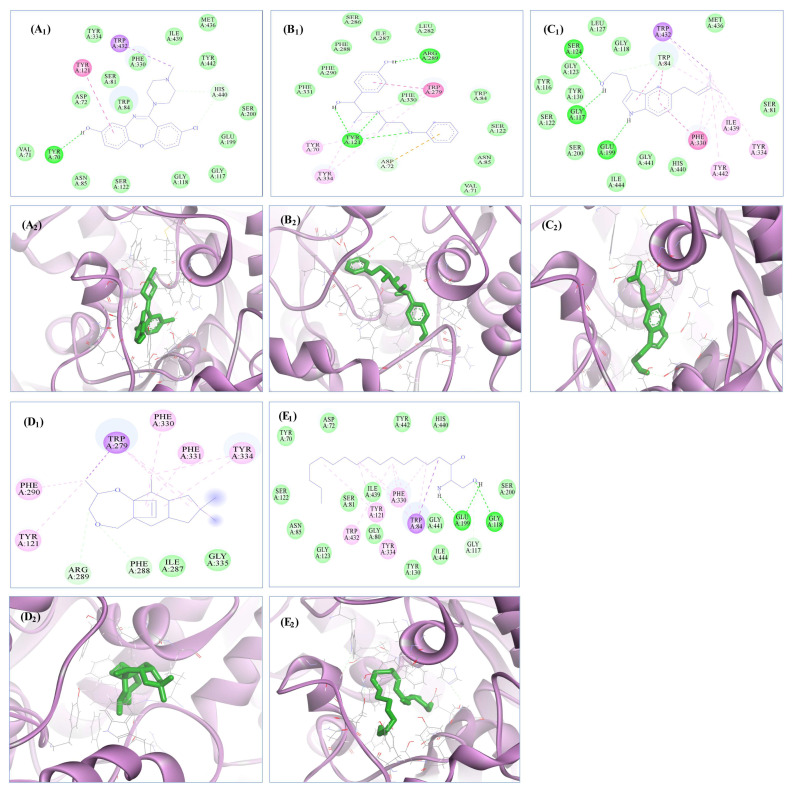
In silico binding patterns of 8-Hydroxyloxapine, Isoxsuprine, MDIE, MHEA, and 2-Aminooctadecaneand against the active pocket of AChE (PDB ID: 1ACJ). (**A_1_**,**A_2_**) Two-dimensional interactions of 8-Hydroxyloxapine within the active pocket of AChE and A_2_ showed the interaction of 8-Hydroxyloxapine with AChE surrounded by α-helix and β-sheet conformations of the active domain of AChE. Similarly, (**B_1_**,**B_2_**) shows Isoxsuprine, (**C_1_**,**C_2_**) MDIE, (**D_1_**,**D_2_**) MHEA, and (**E_1_**,**E_2_**) 2-Aminooctadecaneand.

All the selected compounds were docked with the crystal structure of AChE via AutoDock 4.2, with binding energies (ΔG) ranging from −6.91 to −9.49 kcal/mol. We discovered that the ligands robustly occupied the active pocket of AChE. With binding energies of −9.13 and −9.49 kcal/mol, respectively, 8-Hydroxyloxapine and isoxsuprine showed the greatest affinities for AChE of all the docked ligands. Nineteen amino acid residues (Asn85, Asp72, Glu199, Gly117, Gly118, His440, Ile439, Met436, Phe330, Ser122, Ser200, Ser81, Tyr70, Trp432, Trp84, Tyr121, Tyr334, Tyr442, and Val71), stabilized the 8-hydroxyloxapine/AChE interaction, where one hydrogen bond with Tyr70 and sixteen van der Waals interactions were also noticed. Moreover, Isoxsuprine occupied the AChE active pocket with 17 interacting amino acid residues (Arg28, Asp72, Asn85, Ile287, Phe288, Phe290, Phe330, Phe331, Tyr70, Trp84, Tyr121, Tyr334, Ser122, Trp279, Leu282, Ser286, and Val71), 4 hydrogen bonds with Tyr121 and Arg289 residues, and 12 van der Waals interactions (Table 7 and Figure 7).

**Figure 8:**
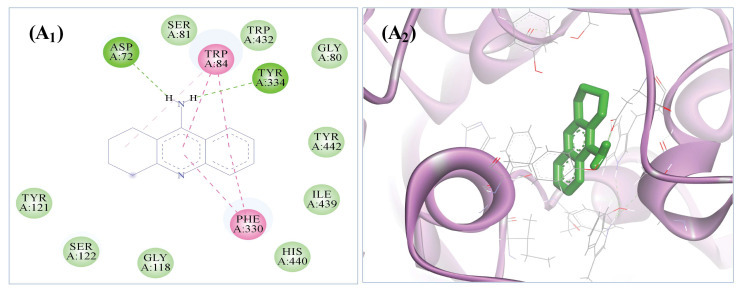
In silico binding patterns of standard drug tacrine (*PubChem* ID: 1935) against the active pocket of AChE (PDB ID: 1ACJ). (**A_1_**,**A_2_**) Two-dimensional interactions of tacrine within the active pocket of AChE and A_2_ panel showed the interaction of tacrine with AChE surrounded by α-helix and β-sheet conformations of the active domain of AChE.

In contrast, the active pocket of AChE was occupied by the tacrine (standard drug) with the binding energy of −8.25 kcal/mol and almost all common residues (i.e., Asp72, Gly80, Gly118, His440, Ile39, Phe330, Ser81, Trp84, Tyr121, Ser122, Tyr334, and Trp432, Tyr442), two hydrogen bonds with Asp72 and Tyr334, and nine van der Waals interactions when compared to the interaction of selected compounds with the AChE binding pocket (Figure 8). All the other selected ligands’ binding energies and amino acid residues interactions with the active pocket of AChE are given in tabular form in Table 7.

The in-silico findings suggest that the presence of the natural compounds in the aqueous fraction of Phoenix dactylifera Khudari cultivar have anti-acetylcholinesterase activity, as they showed strong binding affinity towards the active pocket of AChE when compared to the tacrine.

### 3.10. Molecular Dynamics Simulation (MDS) Analysis

Schrödinger LLC’s Desmond application was utilized to model molecular dynamics for 100 ns [83]. The initial phase of protein and ligand complexes for molecular dynamics modelling was docking studies. Molecular docking studies can forecast the ligand binding state under static conditions. As a result of the fact that docking provides a static representation of a molecule’s binding posture at a protein’s active site [84], MD simulations generally reproduce the motion of atoms over time by integrating Newton’s classical theory of motion, where the physiological environment’s ligand binding state is predicted using simulations [85,86].

#### 3.10.1. RMSD and RMSF Data Analysis

**Figure 9:**
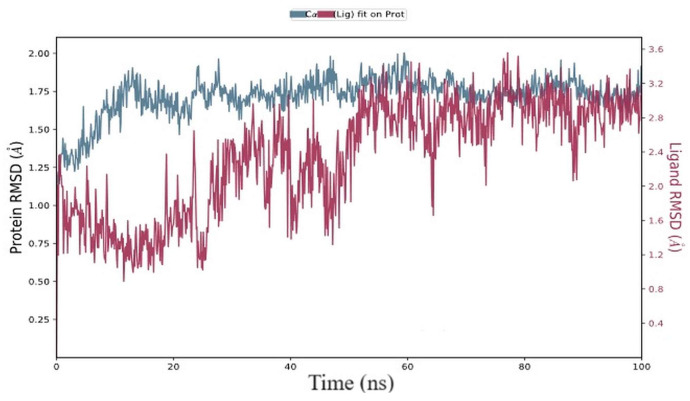
RMSD, i.e., root mean square deviation, of the ligand (2-[5-(1E)-3-methylbuta-1,3-dien-1-yl]-1H-indol-3-ylethanol; *PubChem* CID: 90675402) and the C-alpha atoms of proteins over time. The protein RMSD’s temporal fluctuation is displayed on the left Y-axis. The ligand RMSD’s temporal fluctuation is depicted on the right Y-axis.

As a function of simulation time, the RMSD was calculated by deviating from the structure of the protein–ligand starting posture [67]. In this experiment, we assessed the RMSD of the target proteins (AChE) in the presence of the substance “2-5-[(1E)-3-methylbuta-1,3-dien-1-yl]-1H-indol-3-ylethanol” (*PubChem* CID: 90675402) during a simulation duration of 100 ns (Figure 9). During the initial few seconds and throughout the duration of the simulation, the RMSD of AChE grew. However, in the presence of 90675402, the RMSD of AChE varied from 0 to 50 ns before reaching equilibrium for 50 to 100 ns. The AChE’s average RMSD values were 2.52 0.06 and 3.22 0.25, respectively, during 50 and 100 ns in the presence and absence of 90675402 (Figure 9). Over time, ligand-bound proteins’ C-alpha atoms’ RMSD values varied. The proteins in the complex attained stability at 20 ns, as shown in the RMSD plot. After that, the simulation’s RMSD value fluctuations remained within 1.0 angstrom, which is acceptable. Until 25 ns, the RMSD values of ligands fitted to proteins fluctuated within 1.0 angstrom, after which there was a flip in the ligand mode, with regained stability at 50 ns that remained consistent throughout the simulation.

**Figure 10:**
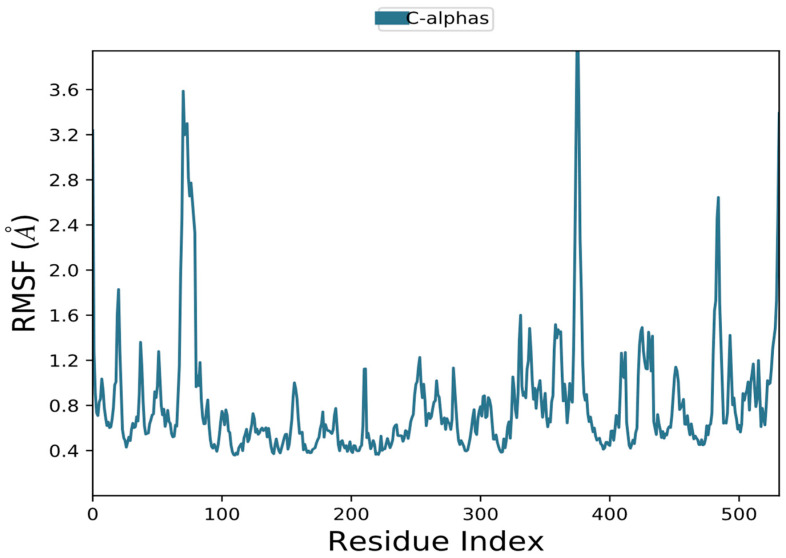
Protein complex residue-by-residue root mean square fluctuation (RMSF).

To detect the local conformational changes in a protein’s side chains brought on by the binding of a ligand, RMSF was frequently monitored [87]. Here, in the presence and absence of the chemical ligand (90675402), the RMSF of AChE was calculated (Figure 10). Proteins’ greater degrees of flexibility at their N- and C-termini cause fluctuations there. In the presence of 90675402, the average RMSF values for AChE were 1.196 and 0.67. The residues with higher maxima belong to loop regions or the N and C termini, according to MD trajectories. Low RMSF values of putative binding residues show the stability of the ligand binding to the protein.

#### 3.10.2. The Solvent-Accessible Surface Area (SASA) and the Gyro-Radius (Rg)

**Figure 11:**
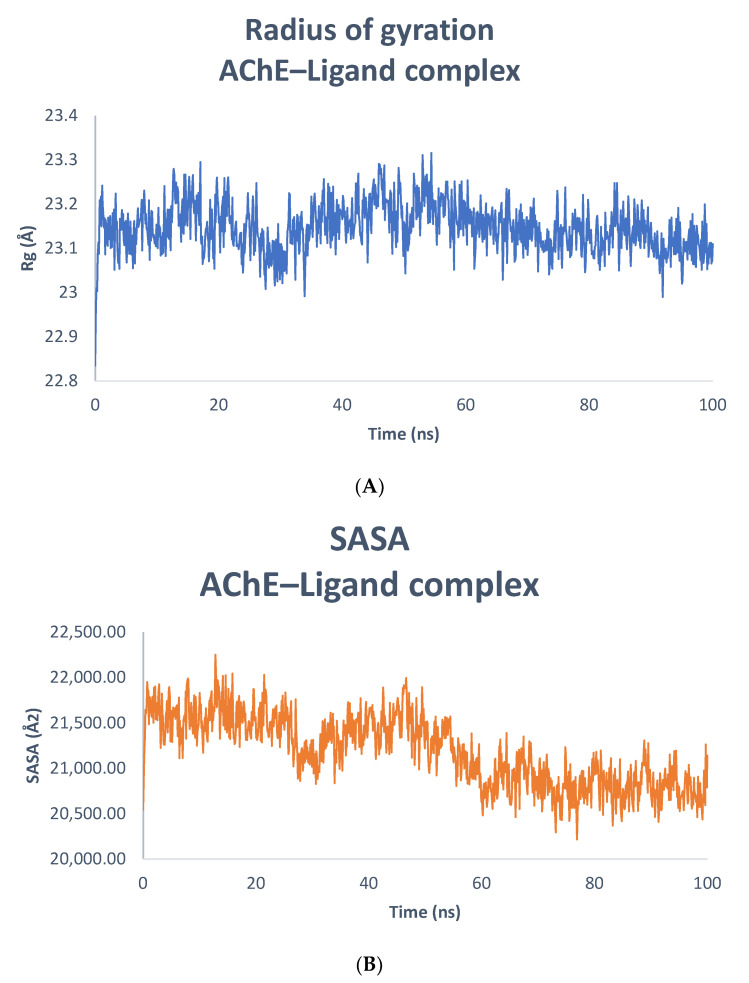
(**A**) Rg of AChE–ligand complex; (**B**) SASA of AChE–ligand complex.

The compactness of the protein–ligand complex was quantified by the radius of gyration, and its exposure to solvent molecules was determined by the solvent-accessible surface area. Each of these traits provides insight into the stability of the protein–ligand combination during the simulation [87]. In this study, the Rg of AChE was determined in the presence of 90675402 during 0–100 ns, as shown in Figure 11A. The Rg of the AChE–90675402 complex varied in the range of 22.84–23.32 Å, with an average value of 23.15 ± 0.06 Å. Similarly, the SASA of AChE was determined in the presence of 90675402 during 0–100 ns (Figure 11B). The SASA of AChE–90675402 complex fluctuated in the range of 20,216.44–22,252.01 Å^2^, with an average value of 21,186.77 ± 384.72 Å^2^. These results demonstrate that changes in the Rg and SASA of the target protein in the presence of 90675402 did not significantly differ, supporting the continued stability of the protein–ligand complexes.

#### 3.10.3. SSEs, i.e., Secondary Structural Elements Study

**Figure 12:**
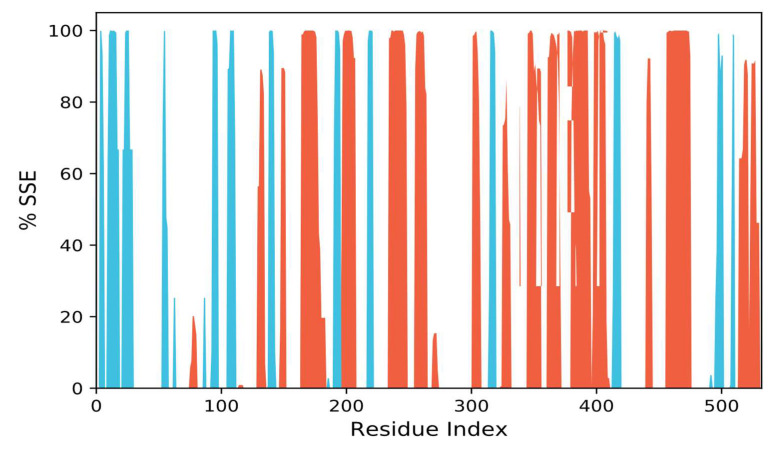
Protein secondary distribution of structural elements by residue index across the complexed protein structures. Alpha helices are denoted by red columns, whereas beta strands are denoted by blue columns.

For the purpose of determining if a protein has undergone any structural changes as a result of ligand binding, it is important to track changes in the secondary structure elements (SSE) of the protein in a protein–ligand complex [88]. The total SSE (α-helix + β-sheet) of AChE in the presence of 90675402 was assessed here as a function of simulation duration (Figure 12). AChE’s SSE was 42.01 2.84% on average when it was in a complex with 90675402 (-helix = 28.63 2.96% and -sheet = 13.38 0.24%). The results reveal a stable protein structure in the complex of protein and ligand since the total SSE for all the targeted proteins did not significantly change when 90675402 was present.

#### 3.10.4. Interactions Analysis

**Figure 13:**
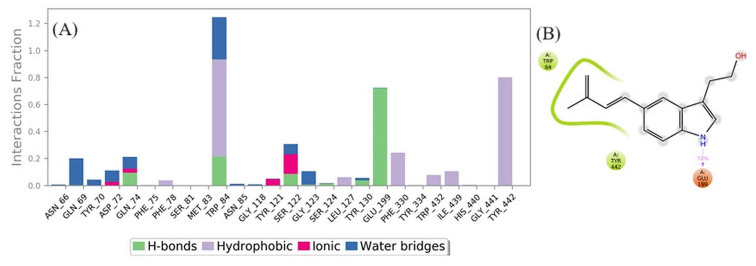
(**A**) Protein–ligand interaction histogram of the complex; (**B**) Residues that interacted with the ligand.

The majority of significant ligand–protein interactions discovered by MD are hydrogen bonds and hydrophobic interactions, as seen in Figure 13. TRP_84 and GLY_442 are the most critical hydrophobic contacts for the complex, although GLU_199 is important for H bonds. The stacked bar graphs were standardized along the trajectory; for instance, a value of 1.0 denotes that the precise interaction was maintained for 100% of the simulation time. Values above 1.0 are achievable simply owing to the possibility of numerous interactions of the same subtype involving a few protein residues and the ligand (Figure 13A,B).

### 3.11. Prime/MM-GBSA Free Energy Analysis

A flexible and reliable way to assess the stabilization of a protein–ligand complex is free energy (Prime/MM-GBSA) computation [89]. After every 10 ns, free energy and its components were calculated using the Prime/MM-GBSA method, and the results are shown in Table 3. For the given complex (AChE–ligand), the average free energy was −52.96 ± 7.46 kcal mol^−1^, dG_Bind_Coulomb −6.51 ± 3.58 kcal mol^−1^, dG_Bind_Covalent 1.67 ± 0.59 kcal mol^−1^, dG_Bind_vdW −35.69 ± 3.12 kcal mol^−1^, dG_Bind_Lipo −36.73 ± 4.38 kcal mol^−1^, and dG_Bind_Hbond −1.30 ± 0.17 kcal mol^−1^. We also observed that the development of a stable complex in the protein–ligand complex was promoted by van der Waals forces, packing contacts, Coulombic forces, and lipophilic interactions (Table 8).

## 4. Conclusions

Cholinesterase inhibition can manage Alzheimer’s disease (AD) by reducing the catalysis of acetylcholine, which ultimately supports the signals transmission and improves the life quality of AD patients. Khudari, a cultivar of *Phoenix dactylifera* that is mostly cultivated in the Al-Qassim region, was found to possess a higher total phenol content and good radical scavenging (DPPH) potential. The Khudari aqueous extract showed the significant inhibition of acetylcholinesterase in a dose-dependent manner, with an enzyme inhibition kinetic study revealing that it exhibits a mixed type of inhibition. Further, for phytochemicals investigation, we used LC/MS analysis, which revealed the presence of eighteen major compounds predicted through the *PubChem* database. Molecular docking and ADMET analysis confirmed that compound, 2-{5-[(1E)-3-methylbuta-1,3-dien-1-yl]-1H-indol-3-yl} ethanol (MDIE) has better binding affinity than tacrine against acetylcholinesterase and was found to be safer, exert considerable aqueous solubility and acceptable LD_50_, and to easily penetrate the BBB. Molecular dynamics analysis of 100 ns and Prime/MM-GBSA supports the stability of the protein–ligand complexes. These findings suggest that aqueous extract of Khudari fruit pulp has significant antioxidant and acetylcholinesterase inhibition potentials and its compound, MDIE, forms a stable confirmation with the target protein, though the fruit of Khudari dates can be a better functional food for the management of Alzheimer’s disease. Further in vivo investigation is required to fully characterize the pharmacokinetic properties, optimization of dose administration, and efficacy of this plant-based natural compound.

## Figures and Tables

**Table 1:** Phytochemicals present in Khudari fruit pulp extracts.

Phytochemicals	Methanolic Extract of KH	Aqueous Extract of KH
Carbohydrates	++	+++
Tannins	+	+
Saponins	-	-
Alkaloids	+	++
Flavonoids	++	+++
Glycosides	+	++
Phenols	++	+++
Terpenoids	+	-
Cardiac glycosides	-	++
Steroids	-	-

(-) not detected; (+) low; (++) moderate; (+++) high concentration.

**Table 2:** Positive-ion mode [M − H]^+^.

Compound ID	Compound Name	Chemical Formula	Match Score	RT	Adduct /Loss	Predicted *m/z*	Matched *m/z*	Fragmentations
73053139	5-(7-Methyloctyl)-1,2,3,4-Tetrahydroquinoline	C_18_H_29_N	0.905	1.71	H^+^/−	260.2373	260.5172	260.517, 260.170, 260.274, 260.309, 261.037
99359	Argvalin	C_11_H_19_N_5_O	0.944	2.67	H^+^/−	238.1662	238.4553	238.04, 238.14, 238.46
90675402	CHEMBL3259981	C_15_H_17_NO	0.917	5.24	H^+^/−	228.1383	228.2916	228.292, 228.049, 227.529, 229.436, 229.818
8182	N-Dodecane	C_12_H_26_	0.859	9.18	H^+^/−	171.2107	171.5068	171.507, 171.195, 171.715, 171.854
10241527	Pyrocoll	C_10_H_6_N_2_O_2_	0.950	11.63	H^+^/−	187.0502	187.3247	187.325, 186.457, 187.776, 188.469
139583859	Methyl hydroxysterpurate ethylidene cetal	C_18_H_30_O_2_	0.851	12.50	H^+^/−	279.2319	279.4917	279.492, 279.249, 279.943, 280.394
139586920	3β-Hydroxy-4β-methylfusida-17(20)(16,21-cis),24-diene	C_30_H_48_O	0.943	14.45	H^+^/−	425.3778	425.6685	425.668, 426.536, 426.778
132579590	Terezine E	C_16_H_22_N_2_O_6_	0.864	19.14	H^+^/−	339.1551	338.8782	338.878, 337.352, 339.052, 339.676, 339.919
10474528	Paeciloquinone D	C_18_H_14_O_9_	0.855	21.09	H^+^/−	375.0711	374.8500	374.850, 374.642, 374.538, 375.093

**Table 3:** Negative-ion mode [M − H]^−^.

Compound ID	Compound Name	Chemical Formula	Match Score	RT	Adduct /Loss	Predicted *m/z*	Matched *m/z*	Fragmentations
45359339	MCULE-1146796980	C_27_H_37_NO_7_	0.989	1.66	-/H^+^	486.2486	486.477	486.477, 486.200, 486.986
8292	Fensulfothion	C_11_H_17_O_4_PS_2_	0.874	1.66	-/H^+^	307.0222	306.9764	306.976, 307.855
43655	8-Hydroxyloxapine	C_18_H_18_ClN_3_O_2_	0.872	1.70	-/H^+^	342.1004	342.3123	342.312, 342.8912
42611990	Gilvsin A	C_30_H_48_O_3_	0.859	2.66	-/H^+^	453.3727	453.3151	453.315, 453.084
222284	Sitosterol	C_29_H_48_O	0.943	3.53	-/H^+^	413.3778	413.3541	413.354
3883	Lansoprazole	C_16_H_14_F_3_N_3_O_2_S	0.971	20.58	-/H^+^	368.0675	368.2130	368.213, 369.045
3783	Isoxsuprine	C_18_H_23_NO_3_	0.967	20.58	-/H^+^	300.1594	300.2700	300.270, 301.195
3126	2-Aminooctadecane	C_18_H_39_NO_2_	0.966	20.58	-/H^+^	300.2897	300.2700	300.270, 301.195
9817839	Dehydroevodiamine	C_19_H_15_N_3_O	0.954	20.66	-/H^+^	300.1131	299.8538	299.854, 300.131

**Table 4:** Pharmacokinetic profiling of the selected compounds predicted in the Khudari aqueous extract.

Compound Name	2-Aminooctadecane	Isoxsuprine	8-Hydroxyloxapine	2-{5-[(1E)-3-methylbuta-1,3-dien-1-yl]-1H-indol-3-yl} Ethanol	Methyl_Hydroxysterpurate Ethylidene Acetal
ID	3126	3783	43655	90675402	139583859
BBB	5.73	2.28	0.49	7.46	1.96
Buffer Solubility mg/L	6.46	541.43	54.65	8.46	66.19
Pure Water Solubility mg/L	2.32	629.45	21.31	25.39	22.11
MDCK	77.07	260.92	97.93	199.83	280.41
Caco2	20.60	15.32	42.70	26.95	57.68
Skin Permeability	−0.77	−3.17	−3.75	−3.07	−2.66
Plasma Protein Binding	100.00	84.22	76.91	83.53	100.00
HIA	87.18	90.24	96.12	91.77	100.00
CYP_2D6_inhibition	Inhibitor	Inhibitor	Non	Non	Non
CYP_2D6_substrate	Substrate	Substrate	Substrate	Non	Non

**Table 5:** Physicochemical properties of the predicted compounds.

Compound	miLogP	M. Wt.	TPSA	Hydrogen Bond Acceptor	Hydrogen Bond Donor	No. of Violations	No. of Rotatable Bond	Volume
3126	5.04	301.51	66.48	3	4	1	16	342.20
3783	2.77	301.39	61.72	4	3	0	7	293.28
43655	3.75	343.81	52.74	5	1	0	1	296.90
90675402	2.98	213.28	36.02	2	2	0	4	209.79
139583859	4.22	278.44	18.47	2	0	0	0	287.92
1935 *	3.05	198.27	38.91	2	2	0	0	191.53

* Standard drug.

**Table 6:** Toxicity assessment of the selected five compounds.

*PubChem* CID	Compound Name	Predicted LD_50_ mg/kg	Predicted Toxicity Class	Hepatotoxicity	Carcinogenicity	Immunotoxicity	Mutagenicity	Cytotoxicity
3126	2-Aminooctadecane	3500	5	NT	NT	NT	NT	NT
3783	Isoxsuprine	200	3	NT	NT	NT	NT	NT
43655	8-Hydroxyloxapine	40	2	NT	NT	T	NT	NT
90675402	2-{5-[(1E)-3-methylbuta-1,3-dien-1-yl]-1H-indol-3-yl}ethanol	1680	4	NT	NT	T	NT	NT
139583859	Methyl Hydroxysterpurate Ethylidene Acetal	7800	6	NT	NT	NT	NT	NT

Note: NT, non-toxic; T, toxic.

**Table 7:** The binding pattern of potential *Phoenix-dactylifera-*derived natural selected compounds against AChE.

S. No.	Compound ID	Compound Name	Binding energy ∆G Kcal/mol	Interacting Amino Acids
1	43655	8-Hydroxyloxapine	−9.49	Asn85, Asp72, Glu199, Gly117, Gly118, His440, Ile439, Met436, Phe330, Ser122, Ser200, Ser81, Tyr70, Trp432, Trp84, Tyr121, Tyr334, Tyr442, Val71
2	3783	Isoxsuprine	−9.13	Arg28, Asp72, Asn85, Ile287, Phe288, Phe290, Phe330, Phe331, Tyr70, Trp84, Tyr121, Tyr334, Ser122, Trp279, Leu282, Ser286, Val71.
3	90675402	2-{5-[(1E)-3-methylbuta-1,3-dien-1-yl]-1H-indol-3-yl} ethanol	−8.67	Gly117, Gly118, Gly123, Gly441, Glu199, His440, Ile444, Ile439, Leu127, Phe330, Ser81, Ser200, Trp84, Tyr116, Ser124, Tyr130, Tyr334 Met436, Trp432, Tyr442,
4	139583859	Methyl hydroxysterpurate ethylidene acetal	−7.91	Try121, Trp279, Ile287, Phe288, Arg289, Phe290, Phe330, Phe331, Tyr334, Gly335.
5	3126	2-Aminooctadecane	−6.91	Asp72, Asn85, Gly80, Gly117, Gly118, Glu199, Gly123, Gly441, His440, Ile439, Ile444, Phe330, Ser122, Ser81, Ser200, Tyr70, Trp84, Tyr121, Tyr130, Tyr334, Tyr442
6	1935*	Tacrine	−8.25	Asp72, Gly80, Gly118, His440, Ile39, Phe330, Ser81, Trp84, Tyr121, Ser122, Tyr334, Trp432, Tyr442

**Table 8:** Before and after simulations, MM-GBSA calculations were performed.

Title/Time	dG_Bind	dG_Bind_Coulomb	dG_Bind_Covalent	dG_Bind_vdW	dG_Bind_Lipo	dG_Bind_Hbond
0 ns	−59.33	−7.61	1.74	−41.71	−41.85	−1.15
10 ns	−40.99	−6.13	1.43	−30.93	−30.56	−1.25
20 ns	−47.96	−8.10	2.40	−32.55	−33.99	−1.20
30 ns	−53.30	2.08	0.63	−36.19	−37.30	−1.07
40 ns	−45.57	−10.64	1.60	−32.09	−29.27	−1.57
50 ns	−48.29	−2.79	1.55	−33.32	−33.75	−1.23
60 ns	−55.58	−8.24	2.63	−34.82	−36.14	−1.46
70 ns	−51.02	−4.76	0.97	−37.19	−37.06	−1.34
80 ns	−57.39	−6.50	2.29	−37.25	−40.67	−1.42
90 ns	−70.20	−11.54	1.93	−37.93	−43.78	−1.52
100 ns	−52.98	−7.37	1.14	−38.68	−39.64	−1.08
AVG	−52.9643581	−6.508649533	1.666593643	−35.69480304	−36.72741552	−1.298206565
SD	7.463234925	3.575755222	0.591036946	3.119070994	4.377846677	0.166159877

## Data Availability

The data presented in this study are available in this article.

## References

[1] Feigin V.L., Nichols E., Alam T., Bannick M.S., Beghi E., Blake N., Culpepper W.J., Dorsey E.R., Elbaz A., Ellenbogen R.G. (2019). Global, Regional, and National Burden of Neurological Disorders, 1990–2016: A Systematic Analysis for the Global Burden of Disease Study 2016. Lancet Neurol..

[2] Geda Y.E., Roberts R.O., Mielke M.M., Knopman D.S., Christianson T.J.H., Pankratz V.S., Boeve B.F., Sochor O., Tangalos E.G., Petersen R.C. (2014). Baseline Neuropsychiatric Symptoms and the Risk of Incident Mild Cognitive Impairment: A Population-Based Study. Am. J. Psychiatry.

[3] Bezoari M.D., Boothe G. (2019). Determination of Potential Multi-Target Inhibitors of Alzheimer’s Disease In Silico. J. Undergrad. Chem. Res..

[4] Leon C., Hill J.S., Wasan K.M. (2005). Potential Role of Acyl-Coenzyme A:Cholesterol Transferase (ACAT) Inhibitors as Hypolipidemic and Antiatherosclerosis Drugs. Pharm. Res..

[5] Glenner G.G., Wong C.W. (1984). Alzheimer’s Disease: Initial Report of the Purification and Characterization of a Novel Cerebrovascular Amyloid Protein. Biochem. Biophys. Res. Commun..

[6] Sarter M., Parikh V. (2005). Choline Transporters, Cholinergic Transmission and Cognition. Nat. Rev. Neurosci..

[7] Rosenberg P.B., Nowrangi M.A., Lyketsos C.G. (2015). Neuropsychiatric Symptoms in Alzheimer’s Disease: What Might Be Associated Brain Circuits?. Mol. Asp. Med..

[8] Saxena M., Dubey R. (2019). Target Enzyme in Alzheimer’s Disease: Acetylcholinesterase Inhibitors. Curr. Top. Med. Chem..

[9] Hampel H., Mesulam M.-M., Cuello A.C., Farlow M.R., Giacobini E., Grossberg G.T., Khachaturian A.S., Vergallo A., Cavedo E., Snyder P.J. (2018). The Cholinergic System in the Pathophysiology and Treatment of Alzheimer’s Disease. Brain.

[10] Dou K.-X., Tan M.-S., Tan C.-C., Cao X.-P., Hou X.-H., Guo Q.-H., Tan L., Mok V., Yu J.-T. (2018). Comparative Safety and Effectiveness of Cholinesterase Inhibitors and Memantine for Alzheimer’s Disease: A Network Meta-Analysis of 41 Randomized Controlled Trials. Alzheimer’s Res. Ther..

[11] Huang L.-K., Chao S.-P., Hu C.-J. (2020). Clinical Trials of New Drugs for Alzheimer Disease. J. Biomed. Sci..

[12] Ahmad J. (2012). Evaluation of Antioxidant and Antimicrobial Activity of Ficus Carica Leaves: An In Vitro Approach. J. Plant Pathol. Microb..

[13] Akhter F., Alvi S.S., Ahmad P., Iqbal D., Alshehri B.M., Khan M.S. (2019). Therapeutic Efficacy of *Boerhaavia diffusa* (Linn.) Root Methanolic Extract in Attenuating Streptozotocin-Induced Diabetes, Diabetes-Linked Hyperlipidemia and Oxidative-Stress in Rats. Biomed. Res. Ther..

[14] Alvi S., Ahmad P., Ishrat M., Iqbal D., Khan S. (2019). Secondary Metabolites from Rosemary (*Rosmarinus officinalis* L.): Structure, Biochemistry and Therapeutic Implications Against Neurodegenerative Diseases. Natural Bio-Active Compounds: Volume 2: Chemistry, Pharmacology and Health Care Practices.

[15] Iqbal D., Khan M.S., Khan M.S., Ahmad S., Srivastava A.K. (2014). An in Vitro and Molecular Informatics Study to Evaluate the Antioxidative and β-Hydroxy-β-Methylglutaryl-CoA Reductase Inhibitory Property of Ficus Virens Ait. Phytother. Res..

[16] Iqbal D., Khan M.S., Khan A., Ahmad S. (2016). Extenuating the Role of Ficus Virens Ait and Its Novel Bioactive Compound on Antioxidant Defense System and Oxidative Damage in Cigarette Smoke Exposed Rats. Biomed. Res. Ther..

[17] Iqbal D., Khan A., A Ansari I., Khan M.S. (2017). Investigating The Role of Novel Bioactive Compound from Ficus Virens Ait on Cigarette Smoke Induced Oxidative Stress and Hyperlipidemia in Rats. Iran J. Pharm. Res..

[18] Jahan S., Ansari U.A., Siddiqui A.J., Iqbal D., Khan J., Banawas S., Alshehri B., Alshahrani M.M., Alsagaby S.A., Redhu N.S. (2022). Nobiletin Ameliorates Cellular Damage and Stress Response and Restores Neuronal Identity Altered by Sodium Arsenate Exposure in Human iPSCs-Derived hNPCs. Pharmaceuticals.

[19] Jana A., Bhattacharjee A., Das S.S., Srivastava A., Choudhury A., Bhattacharjee R., De S., Perveen A., Iqbal D., Gupta P.K. (2022). Molecular Insights into Therapeutic Potentials of Hybrid Compounds Targeting Alzheimer’s Disease. Mol. Neurobiol..

[20] Khatoon A., Khan F., Ahmad N., Shaikh S., Rizvi S.M.D., Shakil S., Al-Qahtani M.H., Abuzenadah A.M., Tabrez S., Ahmed A.B.F. (2018). Silver Nanoparticles from Leaf Extract of Mentha Piperita: Eco-Friendly Synthesis and Effect on Acetylcholinesterase Activity. Life Sci..

[21] Khushtar M., Siddiqui H.H., Dixit R.K., Khan M.S., Iqbal D., Rahman M.A. (2016). Amelioration of Gastric Ulcers Using a Hydro-Alcoholic Extract of Triphala in Indomethacin-Induced Wistar Rats. Eur. J. Integr. Med..

[22] Bhattacharjee R., Das S.S., Biswal S.S., Nath A., Das D., Basu A., Malik S., Kumar L., Kar S., Singh S.K. (2022). Mechanistic Role of HPV-Associated Early Proteins in Cervical Cancer: Molecular Pathways and Targeted Therapeutic Strategies. Crit. Rev. Oncol./Hematol..

[23] Ahmad P., Alvi S.S., Iqbal D., Khan M.S. (2020). Insights into Pharmacological Mechanisms of Polydatin in Targeting Risk Factors-Mediated Atherosclerosis. Life Sci..

[24] El-Seedi H.R., Khalifa S.A.M., Yosri N., Khatib A., Chen L., Saeed A., Efferth T., Verpoorte R. (2019). Plants Mentioned in the Islamic Scriptures (Holy Qur’ân and Ahadith): Traditional Uses and Medicinal Importance in Contemporary Times. J. Ethnopharmacol..

[25] Echegaray N., Pateiro M., Gullón B., Amarowicz R., Misihairabgwi J.M., Lorenzo J.M. (2020). *Phoenix dactylifera* Products in Human Health—A Review. Trends Food Sci. Technol..

[26] Mandal K., Joshi B.C., Dobhal Y. (2022). Phytopharmacological Review on Date Palm (*Phoenix dactylifera*). Indian J. Pharm. Sci..

[27] El-Far A.H., Ragab R.F., Mousa S.A., Al-Khayri J.M., Jain S.M., Johnson D.V. (2021). Date Palm Bioactive Compounds: Nutraceuticals, Functional Nutrients, and Pharmaceuticals. The Date Palm Genome, Volume 2: Omics and Molecular Breeding.

[28] Assirey E.A. (2021). The Chemical Composition, Total Phenolic and Antioxidant Content of Four Date Palm Saudi Cultivars. J. Taibah Univ. Sci..

[29] Amin E., Mohamed E.I.A., Alenezi A.S., Aldwesh M.A., Sebak M., Naguib I.A., Bukhari S.I., Bukhari K., Zaki M.A., Afifi N. (2023). Pattern Recognition of Phytoconstituents and Bioactivities of Date Pit Extracts from Different Cultivars Grown in the Qassim Area. Separations.

[30] Assirey E.A.R. (2015). Nutritional Composition of Fruit of 10 Date Palm (*Phoenix dactylifera* L.) Cultivars Grown in Saudi Arabia. J. Taibah Univ. Sci..

[31] Paulikienė S., Raila A., Žvirdauskienė R., Zvicevičius E. (2019). Application of an Environmentally Friendly Preventive Measure for the Preservation of Fresh Vegetables. J. Food Sci. Technol..

[32] Abdel-Magied N., Ahmed A.G., Abo Zid N. (2018). Possible Ameliorative Effect of Aqueous Extract of Date (*Phoenix dactylifera*) Pits in Rats Exposed to Gamma Radiation. Int. J. Radiat. Biol..

[33] Ipatova O.M., Prozorovskaia N.N., Rusina I.F., Prozorovskiĭ V.N. (2003). Antioxidant properties of a leaf extract from Aronia (*Aronia melanocarba*) containing proanthocyanidins. Biomed. Khim..

[34] Srinivasan M., Rukkumani R., Ram Sudheer A., Menon V.P. (2005). Ferulic Acid, a Natural Protector against Carbon Tetrachloride-Induced Toxicity. Fundam. Clin. Pharmacol..

[35] Subash S., Essa M.M., Braidy N., Awlad-Thani K., Vaishnav R., Al-Adawi S., Al-Asmi A., Guillemin G.J. (2015). Diet Rich in Date Palm Fruits Improves Memory, Learning and Reduces Beta Amyloid in Transgenic Mouse Model of Alzheimer’s Disease. J. Ayurveda Integr. Med..

[36] Pujari R., Vyawahare N., Thakurdesai P. (2014). Neuroprotective and Antioxidant Role of *Phoenix dactylifera* in Permanent Bilateral Common Carotid Occlusion in Rats. J. Acute Dis..

[37] Uddin I., Vandana A.V.A., Kavya G.K.G., Syed Y.H. (2020). Systematic Study on Protective Role of Date Palm (*Phoenix dactylifera* L.) on Central Nervous System Disorders. Ann. Phytomed..

[38] Alsagaby S.A., Iqbal D., Ahmad I., Patel H., Mir S.A., Madkhali Y.A., Oyouni A.A.A., Hawsawi Y.M., Alhumaydhi F.A., Alshehri B. (2022). In silico investigations identified Butyl Xanalterate to competently target CK2α (CSNK2A1) for therapy of chronic lymphocytic leukemia. Sci Rep..

[39] Jabir N.R., Shakil S., Tabrez S., Khan M.S., Rehman M.T., Ahmed B.A. (2021). In Silico Screening of Glycogen Synthase Kinase-3β Targeted Ligands against Acetylcholinesterase and Its Probable Relevance to Alzheimer’s Disease. J. Biomol. Struct. Dyn..

[40] Rehman M.T., AlAjmi M.F., Hussain A., Rather G.M., Khan M.A. (2019). High-Throughput Virtual Screening, Molecular Dynamics Simulation, and Enzyme Kinetics Identified ZINC84525623 as a Potential Inhibitor of NDM-1. Int. J. Mol. Sci..

[41] Shamsi A., Mohammad T., Khan M.S., Shahwan M., Husain F.M., Rehman M.T., Hassan M.I., Ahmad F., Islam A. (2019). Unraveling Binding Mechanism of Alzheimer’s Drug Rivastigmine Tartrate with Human Transferrin: Molecular Docking and Multi-Spectroscopic Approach towards Neurodegenerative Diseases. Biomolecules.

[42] Iqbal D., Rehman M.T., Bin Dukhyil A., Rizvi S.M.D., Al Ajmi M.F., Alshehri B.M., Banawas S., Khan M.S., Alturaiki W., Alsaweed M. (2021). High-Throughput Screening and Molecular Dynamics Simulation of Natural Product-like Compounds against Alzheimer’s Disease through Multitarget Approach. Pharmaceuticals.

[43] Harborne A. (1998). Phytochemical Methods a Guide to Modern Techniques of Plant Analysis.

[44] Singleton V.L., Orthofer R., Lamuela-Raventós R.M. (1999). Analysis of Total Phenols and Other Oxidation Substrates and Antioxidants by Means of Folin-Ciocalteu Reagent. Methods in Enzymology.

[45] Brand-Williams W., Cuvelier M.E., Berset C. (1995). Use of a Free Radical Method to Evaluate Antioxidant Activity. LWT—Food Sci. Technol..

[46] Benzie I.F., Strain J.J. (1996). The Ferric Reducing Ability of Plasma (FRAP) as a Measure of “Antioxidant Power”: The FRAP Assay. Anal. Biochem..

[47] Ellman G.L., Courtney K.D., Andres V., Featherstone R.M. (1961). A New and Rapid Colorimetric Determination of Acetylcholinesterase Activity. Biochem. Pharmacol..

[48] Iqbal D., Khan M.S., Waiz M., Rehman M.T., Alaidarous M., Jamal A., Alothaim A.S., AlAjmi M.F., Alshehri B.M., Banawas S. (2021). Exploring the Binding Pattern of Geraniol with Acetylcholinesterase through In Silico Docking, Molecular Dynamics Simulation, and In Vitro Enzyme Inhibition Kinetics Studies. Cells.

[49] Kim S., Chen J., Cheng T., Gindulyte A., He J., He S., Li Q., Shoemaker B.A., Thiessen P.A., Yu B. (2021). PubChem in 2021: New Data Content and Improved Web Interfaces. Nucleic Acids Res..

[50] Burley S.K., Bhikadiya C., Bi C., Bittrich S., Chen L., Crichlow G.V., Christie C.H., Dalenberg K., Di Costanzo L., Duarte J.M. (2021). RCSB Protein Data Bank: Powerful New Tools for Exploring 3D Structures of Biological Macromolecules for Basic and Applied Research and Education in Fundamental Biology, Biomedicine, Biotechnology, Bioengineering and Energy Sciences. Nucleic Acids Res..

[51] Jiménez J., Doerr S., Martínez-Rosell G., Rose A.S., De Fabritiis G. (2017). DeepSite: Protein-Binding Site Predictor Using 3D-Convolutional Neural Networks. Bioinformatics.

[52] Forli S., Huey R., Pique M.E., Sanner M.F., Goodsell D.S., Olson A.J. (2016). Computational Protein–Ligand Docking and Virtual Drug Screening with the AutoDock Suite. Nat. Protoc..

[53] Morris G.M., Huey R., Lindstrom W., Sanner M.F., Belew R.K., Goodsell D.S., Olson A.J. (2009). AutoDock4 and AutoDockTools4: Automated Docking with Selective Receptor Flexibility. J. Comput. Chem..

[54] Alvi S.S., Ansari I.A., Khan I., Iqbal J., Khan M.S. (2017). Potential Role of Lycopene in Targeting Proprotein Convertase Subtilisin/Kexin Type-9 to Combat Hypercholesterolemia. Free Radic. Biol. Med..

[55] Trott O., Olson A.J. (2010). AutoDock Vina: Improving the Speed and Accuracy of Docking with a New Scoring Function, Efficient Optimization and Multithreading. J. Comput. Chem..

[56] Iqbal D., Rizvi S.M.D., Rehman M.T., Khan M.S., Bin Dukhyil A., AlAjmi M.F., Alshehri B.M., Banawas S., Zia Q., Alsaweed M. (2022). Soyasapogenol-B as a Potential Multitarget Therapeutic Agent for Neurodegenerative Disorders: Molecular Docking and Dynamics Study. Entropy.

[57] Du X., Li Y., Xia Y.-L., Ai S.-M., Liang J., Sang P., Ji X.-L., Liu S.-Q. (2016). Insights into Protein-Ligand Interactions: Mechanisms, Models, and Methods. Int. J. Mol. Sci..

[58] Daina A., Michielin O., Zoete V. (2017). SwissADME: A Free Web Tool to Evaluate Pharmacokinetics, Drug-Likeness and Medicinal Chemistry Friendliness of Small Molecules. Sci. Rep..

[59] Shakil S. (2021). Molecular Interaction of Inhibitors with Human Brain Butyrylcholinesterase. EXCLI J..

[60] Banerjee P., Eckert A.O., Schrey A.K., Preissner R. (2018). ProTox-II: A Webserver for the Prediction of Toxicity of Chemicals. Nucleic Acids Res..

[61] Shivakumar D., Williams J., Wu Y., Damm W., Shelley J., Sherman W. (2010). Prediction of Absolute Solvation Free Energies Using Molecular Dynamics Free Energy Perturbation and the OPLS Force Field. J. Chem. Theory Comput..

[62] Koul B., Farooq U., Yadav D., Song M. (2023). Phytochemicals: A Promising Alternative for the Prevention of Alzheimer’s Disease. Life.

[63] Sawikr Y., Yarla N.S., Peluso I., Kamal M.A., Aliev G., Bishayee A. (2017). Neuroinflammation in Alzheimer’s Disease: The Preventive and Therapeutic Potential of Polyphenolic Nutraceuticals. Adv. Protein Chem. Struct. Biol..

[64] Shaw F.H., Bentley G.A. (1953). The Pharmacology of Some New Anti-Cholinesterases. Aust. J. Exp. Biol. Med. Sci..

[65] Marquis J.K. (1990). Pharmacological Significance of Acetylcholinesterase Inhibition by Tetrahydroaminoacridine. Biochem. Pharmacol..

[66] Pietsch M., Christian L., Inhester T., Petzold S., Gütschow M. (2009). Kinetics of Inhibition of Acetylcholinesterase in the Presence of Acetonitrile. FEBS J..

[67] Farag M.A., Gad H.A., Heiss A.G., Wessjohann L.A. (2014). Metabolomics Driven Analysis of Six Nigella Species Seeds via UPLC-qTOF-MS and GC–MS Coupled to Chemometrics. Food Chem..

[68] Schneider P., Walters W.P., Plowright A.T., Sieroka N., Listgarten J., Goodnow R.A., Fisher J., Jansen J.M., Duca J.S., Rush T.S. (2020). Rethinking Drug Design in the Artificial Intelligence Era. Nat. Rev. Drug Discov..

[69] Daina A., Zoete V. (2016). A BOILED-Egg To Predict Gastrointestinal Absorption and Brain Penetration of Small Molecules. ChemMedChem.

[70] Ma X.L., Chen C., Yang J. (2005). Predictive Model of Blood-Brain Barrier Penetration of Organic Compounds. Acta Pharmacol. Sin..

[71] Leão R.P., Cruz J.V., da Costa G.V., Cruz J.N., Ferreira E.F.B., Silva R.C., de Lima L.R., Borges R.S., Dos Santos G.B., Santos C.B.R. (2020). Identification of New Rofecoxib-Based Cyclooxygenase-2 Inhibitors: A Bioinformatics Approach. Pharmaceuticals.

[72] Bittermann K., Goss K.U. (2017). Predicting Apparent Passive Permeability of Caco-2 and MDCK Cell-Monolayers: A Mechanistic Model. PLoS ONE.

[73] Volpe D.A. (2008). Variability in Caco-2 and MDCK Cell-Based Intestinal Permeability Assays. J. Pharm. Sci..

[74] Yamashita S., Furubayashi T., Kataoka M., Sakane T., Sezaki H., Tokuda H. (2000). Optimized Conditions for Prediction of Intestinal Drug Permeability Using Caco-2 Cells. Eur. J. Pharm. Sci..

[75] Chen C.-P., Chen C.-C., Huang C.-W., Chang Y.-C. (2018). Evaluating Molecular Properties Involved in Transport of Small Molecules in Stratum Corneum: A Quantitative Structure-Activity Relationship for Skin Permeability. Molecules.

[76] Roberts J.A., Pea F., Lipman J. (2013). The Clinical Relevance of Plasma Protein Binding Changes. Clin. Pharmacokinet..

[77] Gurevich K.G. (2013). Effect of Blood Protein Concentrations on Drug-Dosing Regimes: Practical Guidance. Theor. Biol. Med. Model..

[78] Wang B., Yang L.P., Zhang X.Z., Huang S.Q., Bartlam M., Zhou S.F. (2009). New Insights into the Structural Characteristics and Functional Relevance of the Human Cytochrome P450 2D6 Enzyme Structural Features of CYP2D6 B. Wang et Al. Drug Metab. Rev..

[79] Zanger U.M., Schwab M. (2013). Cytochrome P450 Enzymes in Drug Metabolism: Regulation of Gene Expression, Enzyme Activities, and Impact of Genetic Variation. Pharmacol. Ther..

[80] Zhao Y.H., Le J., Abraham M.H., Hersey A., Eddershaw P.J., Luscombe C.N., Boutina D., Beck G., Sherborne B., Cooper I. (2001). Evaluation of Human Intestinal Absorption Data and Subsequent Derivation of a Quantitative Structure—Activity Relationship (QSAR) with the Abraham Descriptors. J. Pharm. Sci..

[81] Hessler G., Baringhaus K.-H. (2018). Artificial Intelligence in Drug Design. Molecules.

[82] Lipinski C.A. (2004). Lead- and Drug-like Compounds: The Rule-of-Five Revolution. Drug Discov Today Technol..

[83] Bowers K.J., Chow E., Xu H., Dror R.O., Eastwood M.P., Gregersen B.A., Klepeis J.L., Kolossvary I., Moraes M.A., Sacerdoti F.D. (2006). Scalable Algorithms for Molecular Dynamics Simulations on Commodity Clusters. Proceedings of the 2006 ACM/IEEE Conference on Supercomputing.

[84] Ferreira L.G., Dos Santos R.N., Oliva G., Andricopulo A.D. (2015). Molecular Docking and Structure-Based Drug Design Strategies. Molecules.

[85] Hildebrand P.W., Rose A.S., Tiemann J.K.S. (2019). Bringing Molecular Dynamics Simulation Data into View. Trends Biochem. Sci..

[86] Rasheed M.A., Iqbal M.N., Saddick S., Ali I., Khan F.S., Kanwal S., Ahmed D., Ibrahim M., Afzal U., Awais M. (2021). Identification of Lead Compounds against Scm (Fms10) in Enterococcus Faecium Using Computer Aided Drug Designing. Life.

[87] Adelusi T.I., Oyedele A.-Q.K., Boyenle I.D., Ogunlana A.T., Adeyemi R.O., Ukachi C.D., Idris M.O., Olaoba O.T., Adedotun I.O., Kolawole O.E. (2022). Molecular Modeling in Drug Discovery. Inform. Med. Unlocked.

[88] Karplus M., Petsko G.A. (1990). Molecular Dynamics Simulations in Biology. Nature.

[89] Genheden S., Ryde U. (2015). The MM/PBSA and MM/GBSA Methods to Estimate Ligand-Binding Affinities. Expert Opin. Drug Discov..

